# A simplified empirical model to estimate oxygen relaxivity at different magnetic fields

**DOI:** 10.1002/nbm.4625

**Published:** 2021-10-02

**Authors:** Emma Bluemke, Eleanor Stride, Daniel Peter Bulte

**Affiliations:** ^1^ Institute of Biomedical Engineering, Department of Engineering Sciences University of Oxford UK

**Keywords:** longitudinal relaxation, oxygen, *R*_1_, relaxivity, vitreous, water

## Abstract

The change in longitudinal relaxation rate (*R*_1_) produced by oxygen has been used as a means of inferring oxygenation levels in magnetic resonance imaging in numerous applications. The relationship between oxygen partial pressure (pO_2_) and *R*_1_ is linear and reproducible, and the slope represents the relaxivity of oxygen (*r*_1Ox_) in that material. However, there is considerable variability in the values of *r*_1Ox_ reported, and they have been shown to vary by field strength and temperature. Therefore, we have compiled 28 reported empirical values of the relaxivity of oxygen as a resource for researchers. Furthermore, we provide an empirical model for estimating the relaxivity of oxygen in water, saline, plasma, and vitreous fluids, accounting for magnetic field strength and temperature. The model agrees well (*R* ^2^ = 0.93) with the data gathered from the literature for fields ranging from 0.011 to 8.45 T and temperatures of 21‐40 °C. This provides a useful resource for researchers seeking to quantify pO_2_ in simple fluids in their studies, such as water and saline phantoms, or bodily fluids such as vitreous fluids, cerebrospinal fluids, and amniotic fluids.

## Graphical abstract

We have compiled 28 reported empirical values of the relaxivity of oxygen and provide an empirical model for estimating the relaxivity of oxygen in water, saline, plasma, and vitreous fluids, accounting for magnetic field strength and temperature. The model agrees well (*R* ^2^ = 0.93) with the data gathered from literature, ranging from 0.011 to 8.45 T and 21 to 40 °C.

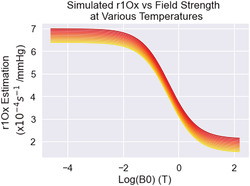

**Abbreviations**
AIC: Akaike information criterion
*B*_0_
: main static magnetic field in MRI scanner
MSE: mean squared error
pO_2_
: partial pressure of oxygen
*R*_1_
: longitudinal relaxation rate
*r*_1Ox_
: relaxivity of oxygen

## 1 INTRODUCTION

Many researchers have investigated using the paramagnetic relaxivity effect of oxygen on longitudinal relaxation as a means of inferring oxygenation levels for applications ranging from cancer therapy to seawater analysis.^1^, ^2^, ^3^, ^4^, ^5^ For example, measurements of the longitudinal relaxation rate *R*_1_ (1/*T*_1_) have been used to infer oxygen levels in vitreous fluid as a noninvasive alternative to the highly invasive oxygen electrodes used to measure retinal hypoxia,^6^, ^7^, ^8^ bladder urine^9^ and urine in the renal pelvis to create a noninvasive detection of renal dysfunction,^5^ and cerebrospinal fluid,^9^, ^10^ and this relationship between pO_2_ and *R*_1_ is also the basis for oxygen‐enhanced MRI techniques.^11^, ^12^, ^13^ In the linear relationship between pO_2_ and *R*_1_, the slope represents the relaxivity of oxygen, or *r*_1Ox_, in that material. Unfortunately, however, there is considerable variability in the values reported for *r*_1Ox_ from empirical measurements, and consequently reliable quantification of pO_2_ from *R*_1_ measurements presents a challenge.

This paper provides a summary of the empirical measurements reported in the literature, investigating the relationship between *R*_1_ and the partial pressure of oxygen in phantoms, saline and water solutions, and vitreous fluid. These experiments have been performed using different equipment and field strengths and reported with a variety of units. For consistency, therefore, all *T*_1_ values will be reported in ms, *R*_1_ in s^−1^, and relaxivity in s^−1^/mmHg oxygen, with the corresponding field strength, temperature and material specified where these data are available. We then propose a simplified empirical model for estimating *r*_1Ox_ in water, saline, plasma, and vitreous fluids based on these reported literature values and report the resulting model parameters. Our aim is to provide a useful review and tool for researchers seeking to quantify pO_2_ in simple fluids, such as water and saline phantoms, or bodily fluids such as vitreous fluids, cerebrospinal fluids, and amniotic fluids. This model does not represent the *r*_1Ox_ in blood or tissue, due to the addition of proteins, structure, cells, lipids, and deoxyhemoglobin, which will affect the *R*_1_‐pO_2_ relationship, to be addressed in a separate manuscript.

## 2 METHODS

### 2.1 Model theory

**FIGURE 1:**
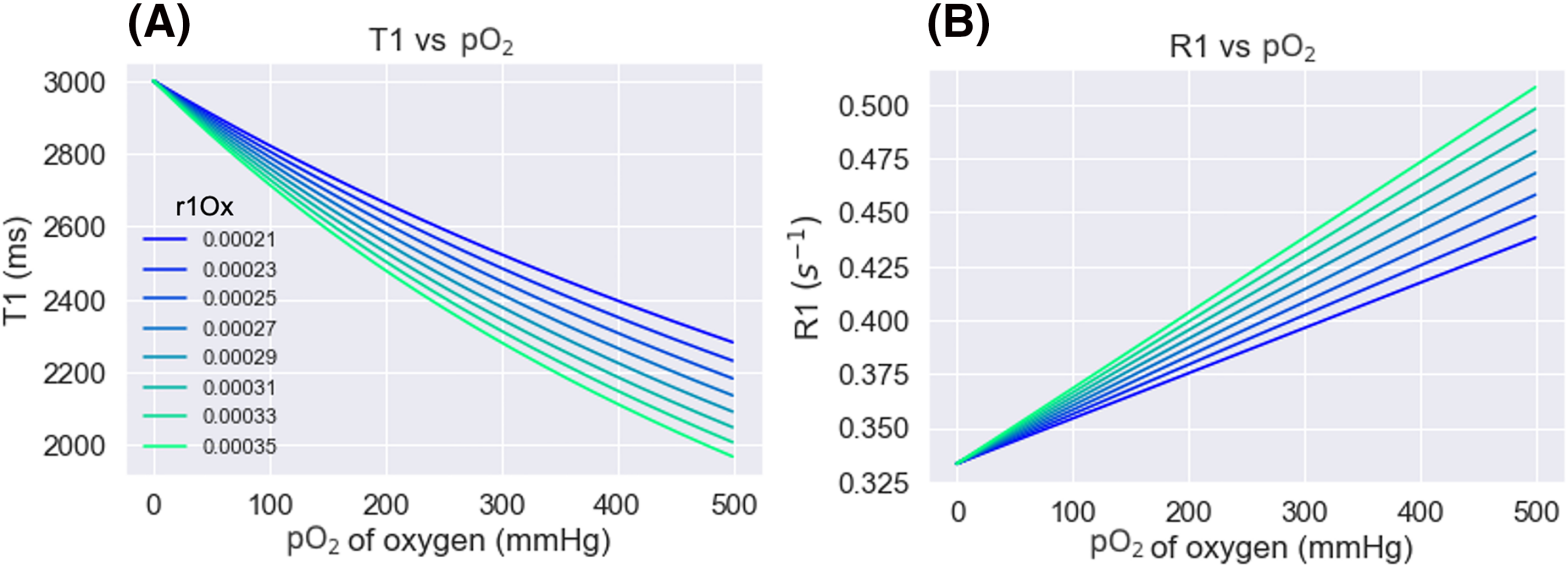
The relationship between *T*_1_ and pO_2_ (A) and *R*_1_ and pO_2_ (B) in a solution, with the initial *T*_1_ of 3000 ms. The values are calculated using a range of *r*_1Ox_ (relaxivity) reported in the literature at 1.5 T, in units s^−1^/mmHg oxygen

*T*_1_ (measured in ms), and its inverse, *R*_1_ (typically reported in s^−1^), have both been used in the literature when reporting the relaxivity effect of oxygen. *R*_1_ is linearly dependent on the concentration of paramagnetic particles,^14^, ^15^ in this case dissolved molecular oxygen in the solution, with the following equation:

(1) $$R_{1\mathrm{Ox}}=R_{1,0}+r_{1\mathrm{Ox}}C$$

where *R*_1Ox_ is the relaxation rate in the solution with oxygen added, *R*_1,0_ is the relaxation rate in the solution without oxygen, *C* is the concentration or partial pressure of oxygen, and *r*_1Ox_ is the relaxivity of oxygen in that solution (whose units depend on the oxygen measurement used in the constant *C*) (shown in Figure 1). Since the partial pressure of oxygen (pO_2_) is a common measurement in biomedicine and clinical applications, in this manuscript, we report *C* as pO_2_ in mmHg and *r*_1Ox_ in s^−1^/mmHg. Conversion factors to other common units such as kPa, Torr, mmol/L, mg/L, and mL/L can be found in Supplementary Table S1.

**FIGURE 2:**
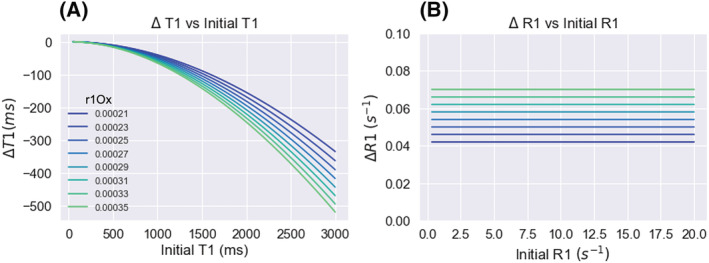
The relationship between Δ*T*_1_ and initial *T*_1_ (A) and Δ*R*_1_ and initial *R*_1_ (B), for a ΔpO_2_ of 200 mmHg. The values are calculated using a range of *r*_1Ox_ (relaxivity) reported in the literature at 1.5 T, in units s^−1^/mmHg oxygen

Changes in both *T*_1_ and *R*_1_ have been used to report changes in pO_2_ in the past.^16^ However, although an increase in oxygen could still qualitatively roughly be inferred from a shortening of *T*_1_, it is important to note that the linear relationship exists with 1/*T*_1_ (*R*_1_)—not *T*_1_—and therefore the change in *T*_1_ caused by oxygen will be dependent on the original *T*_1_ (shown in Figure 2). Therefore, for a quantitative inference of pO_2_ change it is necessary to discuss changes with respect to *R*_1_.

The *r*_1Ox_, or relaxivity of oxygen, is affected by various experimental factors, including field strength. Equations already exist to determine the relationship between field strength and *R*_1_; in 2001, Teng et al.^17^ measured the proton spin‐lattice relaxation rate in water as a function of magnetic field strength at 1 atm of oxygen (approximately 760 mmHg) and found that the magnetic relaxation dispersion due to the paramagnetic contribution from molecular oxygen is well approximated by a Lorentzian shape, for which they proposed the following equation:

(2) $$\frac{1}{T_{1\mathrm{Ox}}}=A\frac{\tau }{1+\tau^{2}{\omega_{s}}^{2}\;}+B\;$$

where *A* and *B* are constants, *ω*_s_ is the electron Larmor frequency, and *τ* is the correlation time for the electron‐nuclear coupling (empirically measured to be 6.8 ± 0.5 ps in water).^17^ One variable of particular interest for medical imaging research is field strength, which can be related to the electron Larmor frequency above (*ω*_s_) by the electron gyromagnetic ratio (*γ*_e_ = 1.76 × 10^11^ rad s^−1^ T^−1^) and Larmor equation *ω*_s_ = *γ*_e_ *B*_0_. By substituting *ω*_s_ = *γ*_e_ *B*_0_ into Equation 2, we can see that

(3) $$\frac{1}{T_{1\mathrm{Ox}}\;}=A\frac{\tau }{1+\tau^{2}{\gamma_{e}}^{2}{B0}^{2}\;}+B.$$

From Equation 1 we know that 1/*T*_1Ox_ is proportional to the relaxivity (*r*_1Ox_), and from Reference 17 we know that the Lorentzian magnetic relaxation dispersion is due to the paramagnetic contribution from molecular oxygen. Therefore, we propose that the relationship between *r*_1Ox_ and field strength will be well approximated by a similar Lorentzian equation with new constants:

(4) $${r1}_{\mathrm{Ox}}=\frac{C_{1}}{1+C_{2}{B0}^{2}\;}+C_{3}$$

where *C*_1_, *C*_2_, and *C*_3_ are new constants, and *B*_0_ is the magnetic field strength (T). It is worth noting that in Equation 4 *C*_1_ will not be equal to *Aτ* as it is in Equation 3, since Equation 3 is calculating *R*_1_ and Equation 4 is calculating *r*_1Ox_—while *R*_1_ and *r*_1Ox_ should be proportional, there are additional multiplying or dividing factors that will be encompassed by *C*_1_.

Finally, the relaxivity of oxygen is also reported to be affected by temperature, as seen in the varied relaxivity measurements reported by Muir et al.^8^ To account for this, we add a fourth constant (*C*_Temp_), which represents the linear slope of relaxivity change due to temperature, resulting in the final equation:

(5) $${r1}_{\mathrm{Ox}}=\frac{C_{1}}{1+C_{2}{B0}^{2}\;}+C_{3}+C_{\text{Temp}}*T.$$

### 2.2 Data collection and analysis

The 28 reported values for oxygen relaxivity were collected from the literature, and all units were converted to s^−1^/mmHg oxygen, shown in Table 1 alongside the field strength and material used in each experiment. If data extraction from graphs was necessary, a digital plot analyzer was used to reliably extract values.^25^

**TABLE 1:** A collection of 28 reported values for oxygen relaxivity from the literature with all units as s^−**1**^/mmHg oxygen alongside the field strength and material used in each experiment. Temperature is indicated where it was reported. The MRI acquisition details from each experiment are listed in Supplementary Table S4

Reference	*r*_1Ox_ (s^−1^/mmHg) × 10^−4^	Field strength (T)	Temp. (°C)	Material
Matsumoto et al., 2006^18^	2.17	4.7	37	Saline
Zaharchuk et al., 2005^10^	2.7	1.5	37	Saline
d'Othée et al., 2003^16^	1.38	8.45	21	Saline
d'Othée et al., 2003^16^	1.90	1.5	21	Saline
Kramer et al., 2013^19^	2.82	1.5	37*	Saline
Kramer et al., 2013^19^	2.21	3	37*	Saline
Simpson et al., 2013^7^	3.6	1.5	35	Saline
Pilkinton et al., 2012^20^	1.61	3	37	Water
Vatnehol et al., 2020^21^	1.9	3	22	Water
Nestle et al., 2003^3^	4.2	0.5	22	Water
Hausser and Noack, 1965^22^	3.72	0.63	22	Water
Zaharchuk et al., 2006^9^	2.49	1.5	37	Water
Graf et al., 1980^23^	6.60	0.011	25	Water
Graf et al., 1980^23^	6.60	0.031	25	Water
Graf et al., 1980^23^	6.23	0.051	25	Water
Graf et al., 1980^23^	6.60	0.137	25	Water
Graf et al., 1980^23^	6.13	0.259	25	Water
Graf et al., 1980^23^	5.18	0.525	25	Water
Graf et al., 1980^23^	3.68	0.713	25	Water
Graf et al., 1980^23^	3.89	0.159	25	Water
Graf et al., 1980^23^	2.74	2.139	25	Water
Graf et al., 1980^23^	2.51	4.387	25	Water
Muir et al., 2013^8^	2.04	3	34	Water
Muir et al., 2013^8^	2.05	3	37	Water
Muir et al., 2013^8^	2.11	3	40	Water
Simpson et al., 2013^7^	3.47	1.5	35	Vitreous fluid
d'Othée et al., 2003^16^	1.11	8.45	21	Plasma (ex vivo)
Hueckel et al, 2000^24^	3.38	1.5	37	Plasma (ex vivo)

* Temperature not reported, assumed to be 37 °C.

**FIGURE 3:**
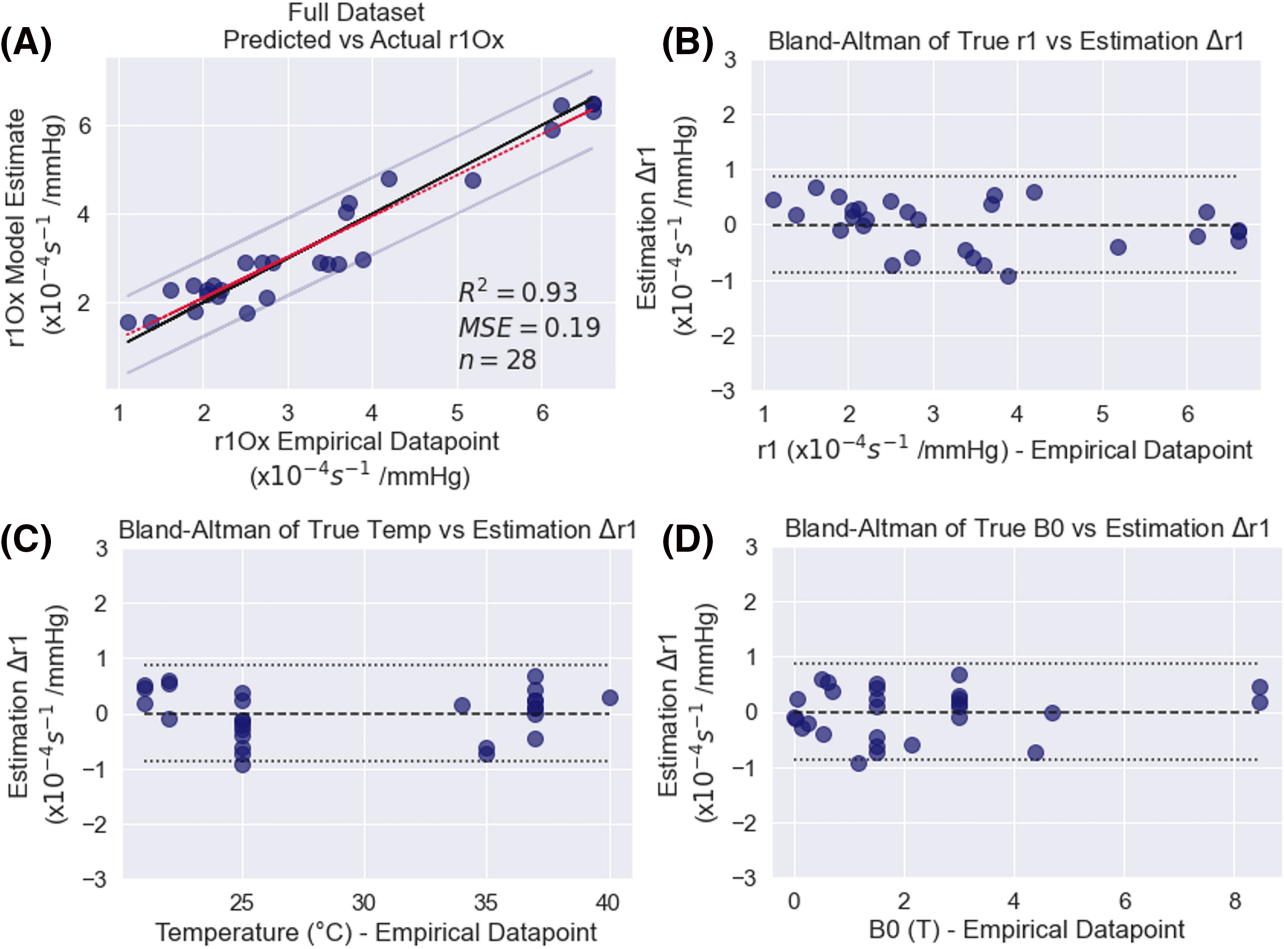
A, The modelled versus measured *r*_1Ox_ values, plotted against the line of equality (solid black line) and a linear regression (red dotted line, *R* ^2^ = 0.93). B, A Bland‐Altman plot showing the difference between the modelled and measured values of *r*_1Ox_. C, D, Bland‐Altman plots for the error in modelled *r*_1Ox_ against temperature (C) and field strength (D) are also shown to examine bias in the model. The horizontal long‐dashed lines show the mean value of Δ*r*_1Ox_ (−0.005 s^−1^ × 10^−4^/mmHg), and the horizontal dotted lines show the limits of agreement (long dashes, calculated from mean(Δ*r*_1Ox_) ± 1.96 SD(Δ*r*_1Ox_))

The SciPy *optimize* function for nonlinear least‐squares fitting was used.^26^ Equation 5 was fitted using a randomized subset of 90% of the 28 literature data points in Table 1, fitting the *B*_0_ and temperature values simultaneously. The dataset was split into randomized subsets for fitting using the sklearn *train_test_fit* function with shuffling.^27^ This process was iterated 1000 times, and the median and 95% confidence interval of the distribution of fitted values for each parameter were used as the final parameters (listed in Table 2). Violin plots showing the distribution of parameter estimates from each iteration are shown in Supplementary Figure S1. Following the model fitting, all four final parameter values were substituted into Equation 5, and the accuracy of the model's predicted *r*_1Ox_ was compared against the ‘true’ *r*1_Ox_ values, shown in Figure 3.

**TABLE 2:** A summary of the final FOUR parameter estimates from the model fitting. Multiply the final model *r*_1Ox_ result by 10^4^ to obtain a relaxivity in units of s^−**1**^/mmHg

Parameter name	Resulting value from fit (95% lower and upper confidence intervals)	Units*
*C*_1_	4.87 (4.70, 5.04)	10^−4^ s^−1^/mmHg
*C*_2_	1.99 (1.59, 2.38)	T^−2^
*C*_3_	0.844 (0.452,1.24)	10^−4^ s^−1^/mmHg
*C*_Temp_	0.0323 (0.0211, 0.0434)	10^−4^ s^−1^/mmHg/°C*

* If using temperature in Kelvin, subtract 273.15 to convert your temperature to °C.

## 3 RESULTS

As shown in Table 1, we found 28 total measurements of *r*_1Ox_: 7 measured in saline solutions,^7^, ^9^, ^16^, ^18^, ^19^ 18 in water,^3^, ^8^, ^9^, ^20^, ^21^, ^22^, ^23^ 1 in vitreous fluid,^7^ and 2 in plasma (ex vivo).^16^, ^24^ The measurements were collected at field strengths ranging from 0.011 to 8.45 T and temperatures ranging from 21 to 40 °C. 12 additional values of *r*_1Ox_, measured in blood (ex vivo^16^, ^24^, ^28^ and in vivo^20^, ^29^) and tissues (lung^30^ and brain^31^), were also found (provided in Supplementary Table S2); however, these were not included in the model fitting because blood and tissues will contain factors not accounted for in this model (see Section 4).

The final values for the four parameters *C*_1_, *C*_2_, *C*_3_, and *C*_Temp_ (with lower and upper 95% confidence intervals) are listed in Table 2. The predicted versus true *r*_1Ox_ values are plotted against the line of equality (solid black line) and a linear regression (dotted line, *R* ^2^ = 0.93) in Figure 3A, with a final mean squared error (MSE) of 0.19 × 10^−4^ (s^−1^/mmHg)^2^. To examine bias in the model with respect to *r*_1Ox_, *B*_0_, and temperaturevariables, Bland‐Altman plots are provided in Figure 3B‐D. The performance of the model on the subsets of water measurements only (*R* ^2^ = 0.94) and saline measurements only (*R* ^2^ = 0.73) is provided in Supplementary Figure S3.

The modelled versus measured *r*_1Ox_ values from the randomized unseen test set of each iteration is plotted in Supplementary Figure S2A alongside the line of equality and the linear regression (*R* ^2^ = 0.90, MSE = 0.26 (× 10^−4^ s^−1^/mmHg)^2^). The difference between the modelled and measured *r*_1Ox_ values from the randomized unseen test set of each iteration is illustrated as Bland‐Altman plots in Supplementary Figure S2B‐D.

**FIGURE 4:**
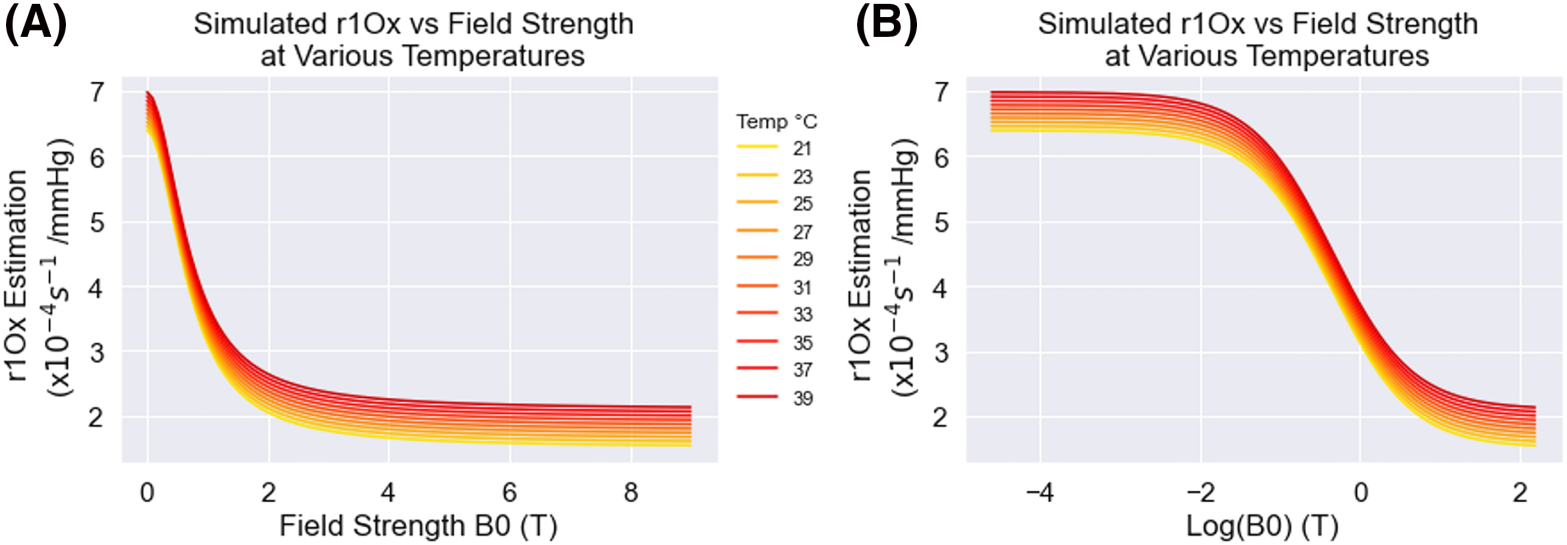
Plots with simulated data to illustrate the behavior of Equation 5 (with the fit parameter values) with respect to *B*_0_ (A) and log(*B*_0_) (B) for a variety of temperatures

**FIGURE 5:**
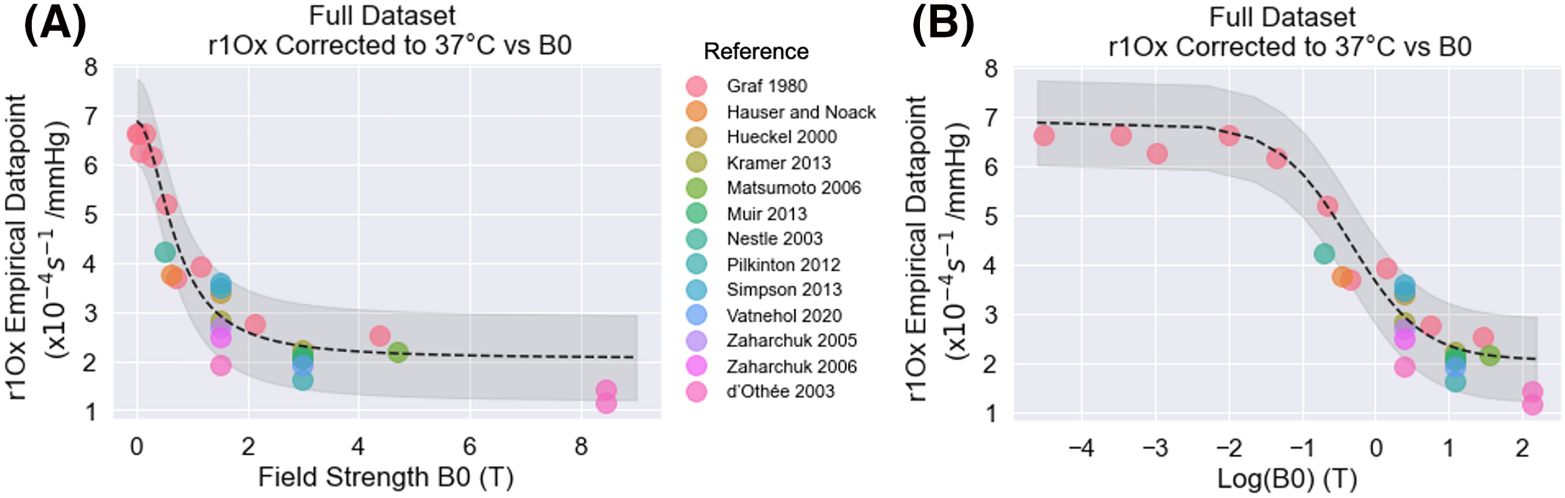
The *r*_1Ox_ of the 28 literature points, plotted against field strength (A) and log(field strength) (B) alongside the *r*_1Ox_ estimated using the new model (black dashed line) at the appropriate *B*_0_ and 37 °C, with the 95% confidence interval shown in grey. For this plot only, each literature value has been linearly corrected to 37 °C using the *C*_Temp_ parameter

To understand the behavior of the resulting model, the effect of varying field strength on *r*_1Ox_ is illustrated using synthetic data under varying temperatures (Figure 4), and the linear effect of varying temperature on *r*_1Ox_ is illustrated using synthetic data under varying field strengths (Supplementary Figure S4). The resulting model prediction is also shown over a scatterplot of the *r*_1Ox_ of the 28 literature points in Figure 5.

Since Equation 5 contains four parameters, the fitting process was repeated for all combinations of fewer parameters (eg removing *C*_Temp_) and the Akaike information criterion (AIC) was calculated for each version of the model—the best‐fit model according to the AIC is the model that explains the greatest amount of variation using the fewest possible independent variables.^32^ The AIC score, *R* ^2^, and MSE results of the different models tested are listed in Supplementary Table S3. The model with all four parameters scored the highest according to the AIC, and was therefore used in this manuscript. Removing only *C*_3_ produced the second‐highest AIC score, and removing only *C*_Temp_ produced the third‐highest AIC score. The resulting predicted versus true *r*_1Ox_ values and parameter distributions from each model are shown in Supplementary Figure S5.

## 4 DISCUSSION

We have compiled empirical measurements of the relaxivity of oxygen over 50 years of MRI research in phantoms, saline and water solutions, plasma, and vitreous fluid, ranging from 0.011 to 8.45 T and 21 to 40 °C. While the reported relaxivity of oxygen varied greatly, we found that in the solutions of water, saline, vitreous fluid, and plasma the variance could largely be explained by the differences in field strength and temperature, and that this variation was well approximated by a Lorentzian function and linear relationship with temperature. Therefore, while the table of reported *r*_1Ox_ empirical measurements can be referred to for future oxygen‐MRI experiments, the *r*_1Ox_ can also be estimated using the proposed simplified model for estimating the *r*_1Ox_ in water, saline, plasma, and vitreous fluids that agrees well with the empirical measurements.

The relationship between longitudinal relaxation and the paramagnetism of oxygen has a long history in NMR, and there is a large body of both theoretical and empirical work.^1^, ^14^, ^15^, ^33^, ^34^, ^35^, ^36^, ^37^ For materials containing paramagnetic contrast agents, there can be complex relationships between *R*_1_ and field strength, and these relationships are affected by various factors inherent to the contrast agent.^38^ This relationship between contrast agent relaxivity and the magnetic field is important, as it can obscure the reproducibility of MRI‐oxygen experiments if performed at different field strengths and temperatures.

The physical mechanisms that explain the relationship between field strength and relaxivity are complex, and although there are sophisticated explanations for specific contrast agents,^39^, ^40^, ^41^, ^42^ much of the modelling of this relaxivity relies on empirical measurements.^43^ While there has been previous evidence for modelling relaxivity‐*B*_0_ relationships as linear^44^ or logarithmic,^45^ one major limitation is that the majority of relaxivity measurements of contrast agents are performed at only two field strengths (1.5 and 3 T), which makes it difficult to properly describe the true relationship from empirical results. Interestingly, to address this issue, an experiment by Chou et al measured the relaxivity of a gadolinium‐based contrast agent at a large range of field strengths, 0‐12 T, revealing a curve that peaks around 1‐2 T and drops off (in a Lorentzian shape) as field strength increases.^39^ While these data are from a gadolinium‐based contrast agent rather than oxygen, they suggest that the relationship of oxygen relaxivity and magnetic field strength may also follow a more complex curve than simply linear, or logarithmic. Therefore, for any modelling of relaxivity, it is important to state the range of field strengths over which the model is valid. The lowest field strength used to fit this model was 0.011 T, and below this field strength it is likely that the *r*_1Ox_ curves back down to zero as field strength approaches zero, in a similar manner to the pattern seen in Figure 4 of Chou et al,^39^ which we have reproduced in Supplementary Figure S6.

### 4.1 Limitations

The relaxivity values collected were from experiments performed over a timespan of five decades, with a huge variation in experimental equipment and temperature measurement techniques. Experimental measurement of *r*_1Ox_ can be difficult even within a relatively simple system such as water, because the measurement accuracy depends on the proper selection of acquisition protocols and parameters (ie repetition and inversion times). For convenience, acquisition details from each experiment used in developing the present model are listed in Supplementary Table S4. MRI technology has advanced significantly since the measurements made in the 1980s, which account for the measurements made below 0.5 T, and low‐field systems usually also suffer from poor signal to noise ratio. In addition, the values have often been extracted from original plots, some hand drawn, which is a source of error, and converted from the various original units to s^−1^/mmHg, which can be another source of error. Finally, one experiment did not report the temperature of the solution during the experiment, and it was assumed to be 37 °C. These limitations inevitably represent a large source of potential error in the accuracy of this model. It is hoped, however, that this can be addressed as the NMR community produces new measurements of *r*_1Ox_ at a range of field strengths and temperatures. As a future direction of this work, we have hosted the open‐source model code and current *r*_1Ox_ measurements on GitHub (github.com/BulteGroup/OxygenRelaxivityModel) and invite the NMR community to share new *r*_1Ox_ measurement results and refit the model to improve the accuracy and enhance the utility of the model.

Furthermore, for the fitting of this model, we have combined values from water, saline, plasma, and vitreous fluids. In reality, there are factors that would cause the relaxivity in these solutions to differ, even amongst saline solutions with different compositions and concentrations. The decision to combine them was due to a lack of sufficient data points at a range of field strengths and temperatures in each solution; however, this is a considerable limitation, as it seems to fit more accurately to the water samples (*R* ^2^ = 0.94) than saline samples (*R* ^2^ = 0.73) alone (Supplementary Figure S3). Most importantly, values from the vitreous fluid and plasma would ideally be fit with separate models, as there are extra proteins in the plasma and vitreous fluid that have been shown to decrease the relaxivity slightly.^7^, ^16^ However, this simply was not possible due to a lack of available data points in the literature. As above, we very much hope that this issue will be addressed as new data are acquired by future researchers.

Finally, this model does not represent the *r*_1Ox_ in blood and tissues, where the addition of proteins, lipids, and deoxyhemoglobin will affect the *R*_1_‐pO_2_ relationship; however, we have created a separate general model to calculate the *R*_1_ of blood, accounting for hematocrit, oxygen saturation, oxygen partial pressure, and magnetic field strength under hyperoxic conditions.^46^ For convenience, however, we have listed the reported literature values found in tissue and blood in Supplementary Table S4—nine values from blood and three from tissues. For the purpose of this model, only the 28 *r*_1Ox_ values in water, saline, vitreous fluid, and plasma were used.

## 5 CONCLUSION

In conclusion, we have provided an overview of the literature reporting a relationship between longitudinal relaxation and oxygen in phantoms, saline and water solutions, and vitreous fluid ranging from 0.011 to 8.45 T and 21 to 40 °C. In addition, we have provided a simplified model for estimating the *r*_1Ox_ in water, saline, plasma, and vitreous fluids that agrees well (*R* ^2^ = 0.93) with the empirical measurements. We hope that this will provide a useful resource for researchers seeking to quantify pO_2_ in simple fluids, such as water and saline phantoms, or bodily fluids such as vitreous fluids, cerebrospinal fluids, and amniotic fluids.

## Supporting information


**Figure S1:** (A) Violin plots showing the distribution of parameter estimates from each iteration. Y axis units per parameter: C_1_ [x10^−4^ s ^−1^/mmHg], C_2_ [T ^−2^], C_3_ [x10^−4^ s ^−1^/mmHg], and C_Temp_ [x10^−4^ s ^−1^/mmHg/°C]. (B) C_Temp_ displayed separately for better visualization of the distribution.
**Figure S2:** (A) The modelled vs measured r1_Ox_ values from the randomized unseen test set of each iteration, plotted against the line of equality (solid black line) and a linear regression (red dotted line, R^2^ = 0.91). (B) A Bland‐Altman plot showing the difference between the modelled and measured values of r1_Ox_. The horizontal long‐dashed lines show the mean value of Δr1_Ox_ (−0.005 s^−1^x10^−4^/mmHg), and the horizontal dotted lines show the limits of agreement (long dashes, calculated by mean (Δr1_Ox_) ± (1.96xSD(Δr1_Ox_)). Bland‐Altman plots for the error in modelled r1_Ox_ against (C) temperature, and (D) field strength are also shown to examine bias in the model.
**Figure S3:** The modelled vs measured r1_Ox_ values, plotted against the line of equality (solid black line) and a linear regression (red dotted line, R^2^ = 0.93) for the subset of data from water samples only (A) and saline samples only (B). Note the smaller range of values available from saline.
**Figure S4:** A plot with simulated data to illustrate the behaviour of Equation 5 (with the fit parameter values) with respect to temperature and for a variety of magnetic field strengths.
**Figure S5:** The fitting process was repeated for all combinations of fewer parameters, and the Akaike Information Criterion (AIC), R^2^, and MSE was calculated for each version of the model. These are the resulting modelled vs measured r1_Ox_ values, plotted against the line of equality (solid black line) and a linear regression (red dotted line) for each model, and violin plots showing the distribution of parameter estimates from each iteration. Y axis units per parameter: C_1_ [x10^−4^ s ^−1^/mmHg], C_2_ [T ^−2^], C_3_ [x10^−4^ s ^−1^/mmHg], and C_Temp_ [x10^−4^ s ^−1^/mmHg/°C]. The model with all 4 parameters (Model A ‐ see yellow star) scored the highest according to the AIC, and was therefore used in this manuscript. For the AIC, R^2^, and MSE values in table form, please see Supplementary Table S3.
**Figure S6:** Reproduced data Figure 4 of Chou et al (2017). The relaxivity profiles of 5‐nm and 30‐nm gadolinium vanadate nanoparticles with and without Eu‐ion doping are presented as a function of the magnetic field.
**Table S1:** Conversion factors from s^−1^/mmHg to other commonly used oxygen units. Converted using Loligo Systems Online Oxygen Converter (*loligosystems.com/convert‐oxygen‐units*).
**Table S2:** A collection of 12 additional reported values for oxygen relaxivity in blood and tissues from the literature with all units to [s^−1^/mmHg oxygen] alongside the field strength and material used in each experiment. Temperature is indicated where it was reported. For experiments performed in vivo, an average body temperature could be assumed.
**Table S3:** The fitting process was repeated for all combinations of fewer parameters, and the Akaike Information Criterion (AIC), R^2^, and MSE was calculated for each version of the model. The best‐fit model according to the AIC is the model that explains the greatest amount of variation using the fewest possible independent variables. The model with all 4 parameters (Model A) scored the highest according to the AIC, and was therefore used in this manuscript. Removing only C_3_ produced the second‐best AIC score, and removing only C_Temp_ produced the third best AIC score. For the resulting plots from each model, please see Supplementary Figure S5.
Table S4: The acquisition details from each experiment listed in Table 1.

## Data Availability

Data sharing not applicable to this article as no datasets were generated or analysed during the current study.
