# Supplementary material for: A simplified empirical model to estimate oxygen relaxivity at different magnetic fields
Source: NMR Biomed. 2021 Oct 2;35(2):e4625. doi: 10.1002/nbm.4625 (PMC11475509; doi:10.1002/nbm.4625)
Supplement: Supplementary file 1 — Figure S1: (A) Violin plots showing the distribution of parameter estimates from each iteration. Y axis units per parameter: C1 [x10−4 s −1/mmHg], C2 [T −2], C3 [x10−4 s −1/mmHg], and CTemp [x10−4 s −1/mmHg/°C]. (B) CTemp displayed separately for better visualization of the distribution. Figure S2: (A) The modelled vs measured r1Ox values from the randomized unseen test set of each iteration, plotted against the line of equality (solid black line) and a linear regression (red dotted line, R2 = 0.91). (B) A Bland‐Altman plot showing the difference between the modelled and measured values of r1Ox. The horizontal long‐dashed lines show the mean value of Δr1Ox (−0.005 s−1x10^−4^/mmHg), and the horizontal dotted lines show the limits of agreement (long dashes, calculated by mean (Δr1Ox) ± (1.96xSD(Δr1Ox)). Bland‐Altman plots for the error in modelled r1Ox against (C) temperature, and (D) field strength are also shown to examine bias in the model. Figure S3: The modelled vs measured r1Ox values, plotted against the line of equality (solid black line) and a linear regression (red dotted line, R2 = 0.93) for the subset of data from water samples only (A) and saline samples only (B). Note the smaller range of values available from saline. Figure S4: A plot with simulated data to illustrate the behaviour of Equation 5 (with the fit parameter values) with respect to temperature and for a variety of magnetic field strengths. Figure S5: The fitting process was repeated for all combinations of fewer parameters, and the Akaike Information Criterion (AIC), R2, and MSE was calculated for each version of the model. These are the resulting modelled vs measured r1Ox values, plotted against the line of equality (solid black line) and a linear regression (red dotted line) for each model, and violin plots showing the distribution of parameter estimates from each iteration. Y axis units per parameter: C1 [x10−4 s −1/mmHg], C2 [T −2], C3 [x10−4 s −1/mmHg], and CTemp [x10−4 s −1/mmHg/°C]. The [file NBM-35-e4625-s001.docx]

# Supplementary Figures


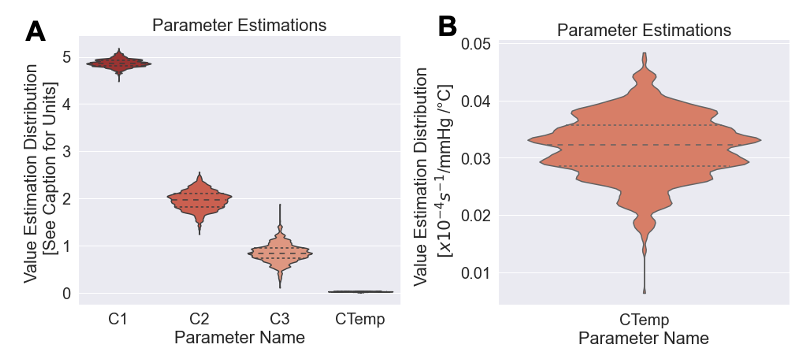


**Supplementary Figure S1**: (A) Violin plots showing the distribution of parameter estimates from each iteration. Y axis units per parameter: C_1_ [x10^-4^  s^−1^/mmHg], C_2_ [T^−2^], C_3_ [x10^-4^  s^−1^/mmHg], and C_Temp_ [x10^-4^ s^−1^/mmHg /°C]. (B) C_Temp_ displayed separately for better visualization of the distribution.


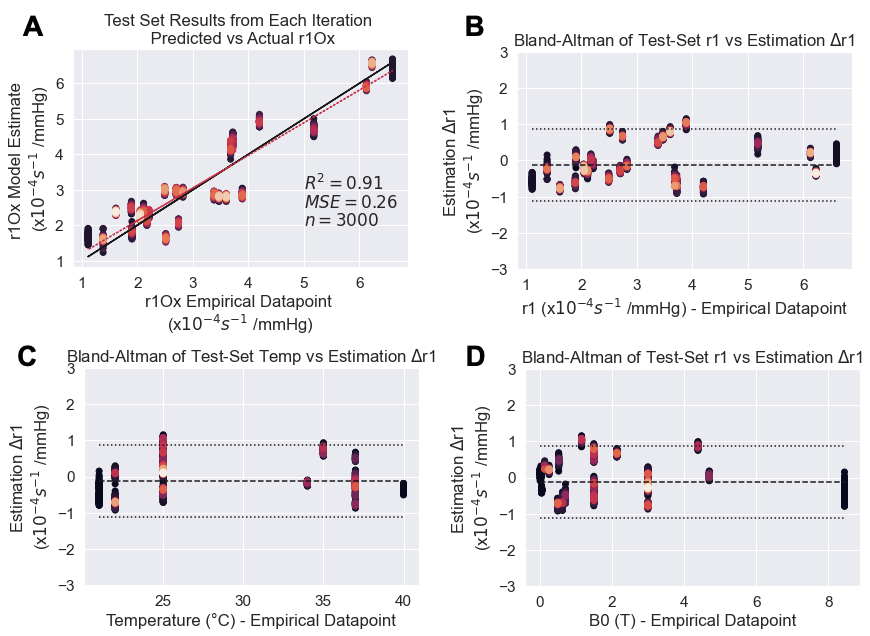


**Supplementary Figure S2:** (A) The modelled vs measured r1_Ox_ values from the randomized unseen test set of each iteration, plotted against the line of equality (solid black line) and a linear regression (red dotted line, R^2^=0.91). (B) A Bland-Altman plot showing the difference between the modelled and measured values of r1_Ox_. The horizontal long-dashed lines show the mean value of Δr1_Ox_ (-0.005 s^-1^x10^-4^/mmHg), and the horizontal dotted lines show the limits of agreement (long dashes, calculated by mean(Δr1_Ox_) ± (1.96xSD(Δr1_Ox_)). Bland-Altman plots for the error in modelled r1_Ox_ against (C) temperature, and (D) field strength are also shown to examine bias in the model.


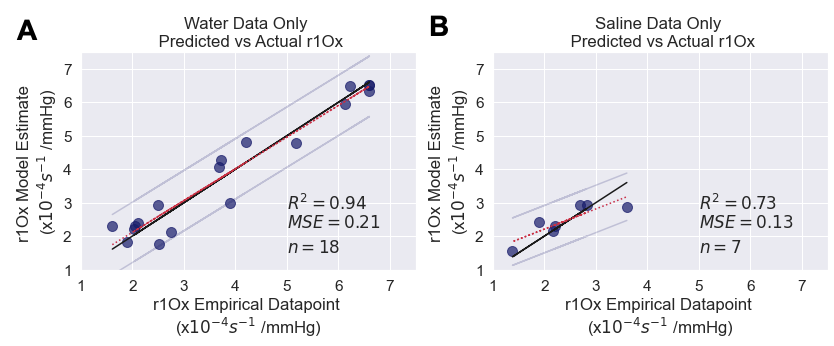


**Supplementary Figure S3:** The modelled vs measured r1_Ox_ values, plotted against the line of equality (solid black line) and a linear regression (red dotted line, R^2^=0.93) for the subset of data from water samples only (A) and saline samples only (B). Note the smaller range of values available from saline.


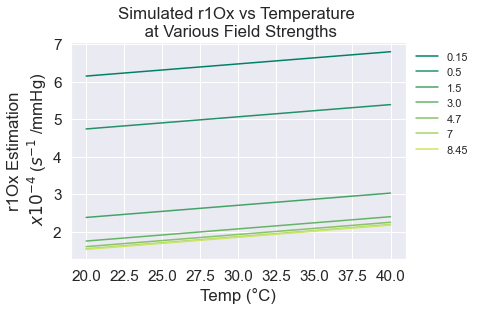


**Supplementary Figure S4:** A plot with simulated data to illustrate the behaviour of Equation 5 (with the fit parameter values) with respect to temperature and for a variety of magnetic field strengths.

**
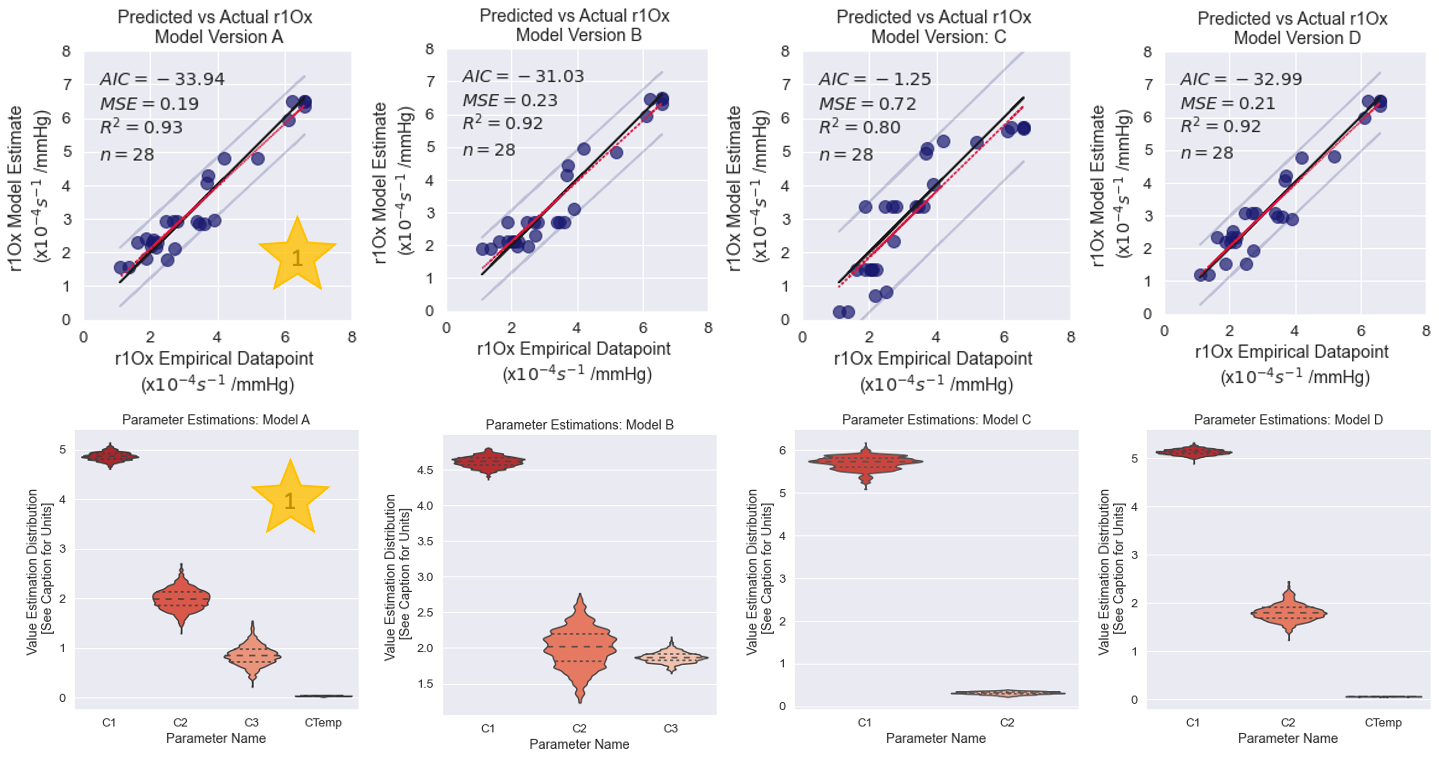
**

**Supplementary Figure S5:** The fitting process was repeated for all combinations of fewer parameters, and the Akaike Information Criterion (AIC), R^2^, and MSE was calculated for each version of the model. These are the resulting modelled vs measured r1_Ox_ values, plotted against the line of equality (solid black line) and a linear regression (red dotted line) for each model, and violin plots showing the distribution of parameter estimates from each iteration. Y axis units per parameter: C_1_ [x10^-4^  s^−1^/mmHg], C_2_ [T^−2^], C_3_ [x10^-4^  s^−1^/mmHg], and C_Temp_ [x10^-4^ s^−1^/mmHg /°C]. The model with all 4 parameters (Model A - see yellow star) scored the highest according to the AIC, and was therefore used in this manuscript. For the AIC, R^2^, and MSE values in table form, please see Supplementary Table S3.


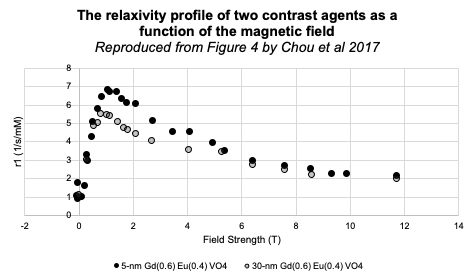


**Supplementary Figure S6:** Reproduced data Figure 4 of Chou et al (2017). The relaxivity profiles of 5-nm and 30-nm gadolinium vanadate nanoparticles with and without Eu-ion doping are presented as a function of the magnetic field.

# Supplementary Tables

##### Supplementary Table S1: Conversion factors from s^-1^/mmHg to other commonly used oxygen units. Converted using Loligo Systems Online Oxygen Converter (*loligosystems.com/convert-oxygen-units*).

| **Commonly used Oxygen Unit** | **Conversion Factor from 1 mmHg Oxygen** | |
| --- | --- | --- |
|  | *Body Temp: 37°C*  *Salinity=0%*  *Atm. Pressure = 1 atm* | *Room Temp: 20°C*  *Salinity=0%*  *Atm. Pressure = 1 atm* |
| **[kPa]** | 1 mmHg = 0.13332 kPa | 1 mmHg = 0.13332 kPa |
| **[Torr]** | 1 mmHg = 1.00000 Torr | 1 mmHg = 1.00000 Torr |
| **[mmol/L]** | 1 mmHg = 0.00140 mmol/L | 1 mmHg = 0.00183 mmol/L |
| **[mg/L]** | 1 mmHg = 0.04470 mg/L | 1 mmHg = 0.05857 mg/L |
| **[mL/L]** | 1 mmHg = 0.03555 mL/L | 1 mmHg = 0.04403 mL/L |

##### Supplementary Table S2: A collection of 12 additional reported values for oxygen relaxivity in blood and tissues from the literature with all units to [s^-1^/mmHg oxygen] alongside the field strength and material used in each experiment. Temperature is indicated where it was reported. For experiments performed in vivo, an average body temperature could be assumed.

| **Reference** | **r1Ox**  **(s^-1^/mmHg) x10^-4^** | **Field strength (T)** | **Temp**  **(°C)** | **Material** |
| --- | --- | --- | --- | --- |
| **BLOOD** | | | | |
| d’Othée et al., 2003 | 2.5 | 8.45 | 21 | Blood (*ex vivo*) |
| d’Othée et al., 2003 | 3.5 | 1.5 | 21 | Blood (*ex vivo*) |
| Tripathi et al., 1984 - Dog 2 | 2.9 | 0.15 | *In vivo* | Blood (left ventricle) |
| Tripathi et al., 1984 - Dog 3 | 3.8 | 0.15 | *In vivo* | Blood (left ventricle) |
| Tripathi et al., 1984 - Dog 4 | 2.5 | 0.15 | *In vivo* | Blood (left ventricle) |
| Tripathi et al., 1984 - Dog 5 | 9.2 | 0.15 | *In vivo* | Blood (left ventricle) |
| Silvennoinen et al., 2003 | 4.1 | 4.7 | 37 | Blood (*ex vivo*) |
| Hueckel et al, 2000 | 4.38 | 1.5 | 37 | Erythrocyte-enriched blood (*ex vivo*) |
| Pilkinton et al., 2012 | 1.44 | 3 | *In vivo* | Blood |
| **TISSUES** | | | | |
| Campbell-Washburn et al., 2019 | 4.7 | 0.55 | *In vivo* | Lung tissue |
| Campbell-Washburn et al., 2019 | 3.1 | 1.5 | *In vivo* | Lung tissue |
| Beeman et al., 2016 | 9.0 | 4.7 | *In vivo* | Brain tissue |

**Supplementary Table S3:** The fitting process was repeated for all combinations of fewer parameters, and the Akaike Information Criterion (AIC), R^2^, and MSE was calculated for each version of the model. The best-fit model according to the AIC is the model that explains the greatest amount of variation using the fewest possible independent variables. The model with all 4 parameters (Model A) scored the highest according to the AIC, and was therefore used in this manuscript. Removing only C_3_ produced the second-best AIC score, and removing only C_Temp_ produced the third best AIC score. For the resulting plots from each model, please see Supplementary Figure S5.

| **Equation** | | **Variables included** | **Rank (AIC)** | **K** | **AIC Score** | **R^2^** | **MSE** |
| --- | --- | --- | --- | --- | --- | --- | --- |
| **A** | $r1_{Ox}=\frac{C_{1}}{1 + C_{2}{B0}^{2}} +C_{3} + C_{Temp}*T$ | C_1_, C_2_, C_3_, C_Temp_ | 1 | 2+4=6 | -33.94 | 0.93 | 0.19 |
| **B** | $r1_{Ox}=\frac{C_{1}}{1 + C_{2}{B0}^{2}} +C_{3}$ | C_1_, C_2_, C_3_ | 3 | 2+3=5 | -31.04 | 0.92 | 0.23 |
| **C** | $r1_{Ox}=\frac{C_{1}}{1 + C_{2}{B0}^{2}}$ | C_1_, C_2_ | 4 | 2+2=4 | -1.25 | 0.80 | 0.72 |
| **D** | $r1_{Ox}=\frac{C_{1}}{1 + C_{2}{B0}^{2}} + C_{Temp}*T$ | C_1_, C_2_, C_Temp_ | 2 | 2+3=5 | -32.99 | 0.92 | 0.215 |

#####

#####

##### Supplementary Table S4: The acquisition details from each experiment listed in Table 1.

| **Reference** | **MRI Acquisition Details (Relevant Section)** |
| --- | --- |
| Matsumoto 2006 | For T1 and T2 mapping, spin-echo images were obtained using a multislice multiecho (MSME) sequence with two different TRs (4000 and 800 ms) and a 16-echo train with TE=15 ms. The scantime for the T1 andT2 mapping image set (NEX=1) by the MSME sequence was 10 min. SPGR (also referred to as gradient-echo fast imaging (GEFI); TR=75 ms, TE=3 ms,FA=45°, NEX=8) was employed to observe the T1 effect. |
| Zaharchuk 2005 | Based on a compromise between spatial and temporal resolution established during pilot studies, we chose to measure T1 using a two-point 3D modiﬁed fast inversion recovery (MFIR) method (TR/TE/TI=11 s/140 ms/[3.2 s,none], fast spin-echo [FSE] readout). Additionally, to test the validity of the two-point method, more complete sampling of the IR curve was performed with 10 different TIs (TR/TE/TI = 11s/140 ms/[0.4, 0.8, 1.2, 1.6, 2.0, 2.4, 2.8, 3.2, 4.0, 5.0 s]). T1was measured using a least-squares, three-parameter ﬁtting method. |
| d’Othée et 2003 | With the 8.45-Tesla magnet, calculations of T1 were based on the evolution of the signal intensity (SI) over time (T1 relaxation curve) after stimulation by a T1-weighted inversion recovery (IR) sequence using multiple inversion times (TIs). Nine different TIs were chosen:0.008, 0.05, 0.25, 0.5, 0.8, 1.0, 4.0, 7.0, and 12.0 seconds. A standard broadband probe with a 20-mm diameter radiofrequency coil was used for all measurements. The experimental conditions were such that the temporal resolution was under 3 minutes per T1 measurement. The following parameters were applied: one average, sweep width of 4,990 Hz, matrix size 8,192, and the duration of the 180°pulse was 110 microseconds. The signal intensity values were fitted on MATLAB software according to the equation: I_(t)_=I_(0)_(1–2e^(-TI/T1)^).  On the 1.5-Tesla MRI, we used an inversion recovery sequence with multiple TIs of 0.2, 0.4, 0.8, 1, 2, 4, 5, and 6 seconds. Acquisition parameters were as follows: repetition time=8 seconds, flip angle=8 degrees, slice thickness=8 mm, matrix=64x128, field of view=150x150 mm. |
| Kramer 2013 | An inversion recovery sequence with 20 different inversion times (100 ms-15 s) was used to determine the T1 time, and a spin-echo sequence with a total of 9 different echo times (10 ms-2 s) was used to determine the T2 time. The measured T1 and T2 times made it possible to calculate the relaxivity of oxygen. |
| Simpson 2013 | The scanning protocol used an inversion recovery (IR) true fast imaging with steady-state precession (TrueFISP) imaging sequence that was repeated using 17 inversion times (TI) in the range 0.7 to 30 seconds. The order of the inversion times was initially randomized to mitigate potential drift effects in the scanner. Other TrueFISP parameters were: repetition time =(20 þ TI) seconds, echo time = 1.52 ms, flip angle = 808, matrix =256 x 256, and voxel dimensions = 0.9 x0.9 x 4 mm^3^. |
| Pilkinton 2012 | An inversion-prepared single-shot FSE sequence was used with 10 inversion times (90, 200, 400, 800,1200, 1600, 2400, 4000, 6000, 1000 ms) with a TR=20 s. |
| Vatnehol 2020 | Based on a previous study on in vivo stability [9], two MOLLI schemes of 10(5)5 and 5(3)3 were selected. Table 1 shows selected sequence parameters for both sequences. Parameters relevant for contrast were flip angle=20, TR/TE shortest (2.4/1.07 ms and 2.6/1.2 ms), and minimum TI 178.3 and 196.1 ms for the MOLI 10(5)5 and 5(3)3, respectively. A simulated ECG signal was used for image triggering at a rate of 60 beats per minute. Following localizer images, the sequences for quantitative estimation of T1 were acquired six times for each sequence. |
| Nestle 2003 | The NMR studies were performed in a simple NMR relaxometer MRS 6 (JSI, Ljubljana, Slovenia). |
| Hausser and Noack 1965 | Used an NMR relaxometer. The manuscript is in German, please refer to the original manuscript for NMR details. |
| Zaharchuk 2006 | Images were acquired using the following parameters: T_SR1_ =10 seconds, T_SR2_=3 seconds, TE =200 milliseconds, field  of view (FOV)=20 cm, matrix 320x256, slice thickness =8 mm, receiver bandwidth =31.25 kHz, and echo train length 136. R1 was determined by the iterative method described in Appendix A (Iterative R1 Measurement Using a Dual-Shot  SSFSE Sequence), using five iterations. |
| Graf 1980 | Used an NMR relaxometer. Please refer to the original manuscript for NMR details. |
| Muir 2013 | R1 measurements were made by using a Look-Locker sequence (11) with a spoiled gradient-echo readout, in which data at 26 inversion times were acquired after a single inversion pulse with minimum inversion time of 103 msec; the equal spacing between inversion times was 264 msec. Images were acquired in two shots with the following parameters: repetition time msec/echo time msec, 6.6/3.2; field of view, 100 x 100 mm; matrix, 100 x 100; partial Fourier factor, 0.8; a single 6-mm section; flip angle, 4°; six to nine repetitions; and 20 seconds between repetitions of the inversion pulse. |
| Hueckel 2000 | Tl relaxation times were measured by use of an inversion recovery single shot turbo spin echo sequence (RARE). The imaging parameters were as follows: TE,s = 4.2 ms, inter-echo time = 4.2 ms, slice thickness = 10 mm, FOV = 240 mm x 240 mm. The inversion time (TI) was adjusted to 16 different values in the range between 50 ms and 9000 ms for each Tl measurement.  Calculation of the Tl values was done by a three- parameter-fit corresponding to the following equation: Si (TI) = a + b exp(-TI/Tl). This fit compensates for the influence of imperfect inversion pulses on the calculated Tl values. |

##### 
